# Recurrent selection breeding by dominant male sterility for multiple abiotic stresses tolerant rice cultivars

**DOI:** 10.1007/s10681-017-2055-5

**Published:** 2017-11-09

**Authors:** Yunlong Pang, Kai Chen, Xiaoqian Wang, Jianlong Xu, Jauhar Ali, Zhikang Li

**Affiliations:** 1 Institute of Crop Sciences/National Key Facility for Crop Gene Resources and Genetic Improvement, Chinese Academy of Agricultural Sciences, Beijing, 100081 China; 2 International Rice Research Institute, DAPO Box 7777, Metro Manila, Philippines; 3 State Key Laboratory of Crop Biology, College of Agronomy, Shandong Agricultural University, Taian, 271018 China; 4 Agricultural Genomics Institute, Chinese Academy of Agricultural Sciences, Shenzhen, 518120 China; 5 Shenzhen Institute of Breeding and Innovation, Chinese Academy of Agricultural Sciences, Shenzhen, 518120 China

**Keywords:** Dominant male sterility, Drought, Recurrent selection, Rice, Salinity

## Abstract

Recurrent selection has not been extensively applied in rice breeding practices due to lack of male sterility genes. Recently, a male sterile line (named as ‘Jiabuyu’) controlled by a novel single dominant gene was found, which provides an ideal tool for rice recurrent selection breeding. Here, two different kinds of recurrent selection populations facilitated for outcrossing by the dominant male sterile line ‘Jiabuyu’ were developed, that included one population using 31 abiotic stress tolerance introgression lines with common recipient parent as founders, and the other one using 25 popular restorers lines applied in hybrid breeding as founders. After two cycles of recurrent selection through natural outcrossing, the seeds from male fertile progeny plants were screened for higher grain yield under normal irrigated, drought, and salt-stressed natural field conditions. Finally, we identified 11 promising high-yielding lines under irrigated conditions, 12 drought-tolerant and 12 salt-tolerant lines. Among them, one line gave higher grain yield across all three conditions, three lines yielded high in both irrigated and drought conditions and another three lines gave high yields in both drought and salt-stressed conditions. The present study was a commendable attempt at utilizing recurrent selection population facilitated by dominant male sterile line to improve rice complex traits, which provided valuable lessons for other rice breeders. The developed lines are promising to be rice varieties with high yield, drought and/or salinity tolerances.

## Background

Rice (*Oryza sativa* L.) is one of the most important staple foods feeding more than half of the world’s population. It is estimated that a 50% increase in rice grain yield (GY) may be required by 2050 to keep hunger away (Sheehy et al. 2002). Thus, improving rice yield potential or its yield under stress conditions is the foremost task for rice breeders (Ali et al. 2017). Rice production is adversely affected by numerous biotic and abiotic stresses, including diseases and pests, cold, drought, flooding, heat, and salinity. Among them, drought and salinity are the major abiotic constraints that reduce GY, especially in Asia, whose population grows and consumes approximately 90% of rice (Ali et al. 2006). Drought is the major stress that brings about severe yield reductions in rainfed upland areas across countries in South and Southeast Asia (Kumar et al. 2008). In China, the increasing water scarcity for irrigation threatens rice production even in the irrigated areas, thus creating major concern (Peng et al. 2009). Coastal saline soils cover millions of hectares in the humid regions of South and Southeast Asia, which remain uncultivated, and, whenever rice is planted in such conditions, this results in extremely low GY. The current salinization problem of irrigated paddy soils, especially under huge irrigation dam project command areas in Asia, is directly affecting the stability of rice production in Asia. It is predicted that, over the next 25 years, nearly 30% of the cultivated land will be salinized because of global climate change, mismanaged agricultural practices, and aggravated industrial pollution (Jagadish et al. 2012). Therefore, developing drought- and salt-tolerant cultivars is an important strategy to reduce risk and increase rice productivity (Blumwald and Grover 2006).

Over the past decade, extensive efforts have been devoted to developing drought- and/or salt-tolerant rice cultivars (Ali et al. 2006; Bernier et al. 2008; Kumar et al. 2008; Swamy et al. 2011, 2017; Chai et al. 2013; Swamy and Kumar 2013; Wang et al. 2013; Bimpong et al. 2016; Ali et al. 2017). These cultivars were mostly developed through conventional cross-pedigree breeding and marker-assisted backcross breeding approaches to enhance the ability of modern rice varieties to tolerate drought and salinity stresses by using diverse rice accessions. However, in conventional breeding programs, only a few parents are involved and their use efficiency in rice accessions remains low because of the inefficient cross-pedigree breeding method, resulting in a narrow genetic base of the developed cultivars (Li and Zhang 2013). Further, using such approaches, one cannot improve breeding populations in a continuum to pyramid multiple favorable alleles. Similarly, breeding restorer lines for developing heterotic rice hybrids faces a similar problem. There is a need for developing restorer lines with improved combining ability along with multiple abiotic stress tolerance traits. As higher GY and drought and salinity tolerance are highly complex and quantitative traits that are governed by multiple genes/QTLs, and there are lots of cryptic beneficial alleles (Ali et al. 2006), through conventional cross-pedigree breeding approaches, it is difficult to use and pyramid all of these genes at one time.

Recurrent selection (RS) involving dozens of parents is considered as an ideal breeding approach to steadily improve the level of quantitative traits in a breeding population. The RS breeding approach was first applied in cross-pollinated crops, mainly in maize (Bolaños and Edmeades 1993). For often cross-pollinated and self-pollinated crops, however, RS remains restricted by the hard and inefficient artificial crossing method, which was later resolved by using genetic male sterile (MS) that facilitated the application of RS in often cross-pollinated crops such as cotton (Meredith and Bridge 1973) and sorghum (Doggett 1972), and self-pollinated crops such as soybean (Brim and Stuber 1973; Posadas et al. 2014), wheat (Ramya et al. 2016; Marais et al. 2000), and rice (Grenier et al. 2015; Frouin et al. 2014; Fujimaki 1979). In rice, Virmani et al. (1997) had shown a random mating composite population facilitated by IR36ms having recessive genic male sterility for the improvement of restorers and maintainers in a successful manner. However, being a recessive genic male sterility system, it was cumbersome and inefficient in the management of the RS population. Thus, discovery and use of the MS gene are essential for successful RS breeding in rice. In 2001, a mutant of “Sanming Dominant Genic Male Sterile Rice” was found from an F_2_ population of a cross between SE21S and Basmati370 by the Sanming Institute of Agricultural Science (Huang et al. 2008). It was proven that the male sterility of this mutant was controlled by a dominant gene and it was fine mapped on chromosome 8 (Yang et al. 2012). By multiple backcrosses, this dominant male sterile (DMS) allele was introduced into the genetic background of *Xian* rice cultivar Jiafuzhan (designated as ‘Jiabuyu’) (Yang et al. 2012), which allowed us to use it for RS breeding.

In the present study, the DMS line ‘Jiabuyu’ was used as an outcrossing facilitator and two different types of RS populations were developed. First, the initial population for inbred breeding comprised 31 elite introgression lines and the second population for hybrid breeding comprised 25 restorer lines as founders. The plant progeny materials were used to screen for high yield (HY) and drought and salt tolerance. Later, selected promising lines were evaluated over two rounds of replicated yield trials across two seasons, which were effectively compared with national irrigated and drought- and salt-tolerant varietal checks of the Philippines. The breeding strategy of RS facilitated by DMS for developing multiple stress tolerance is presented and discussed in this article.

## Materials and methods

### Plant materials

**Fig. 1:**
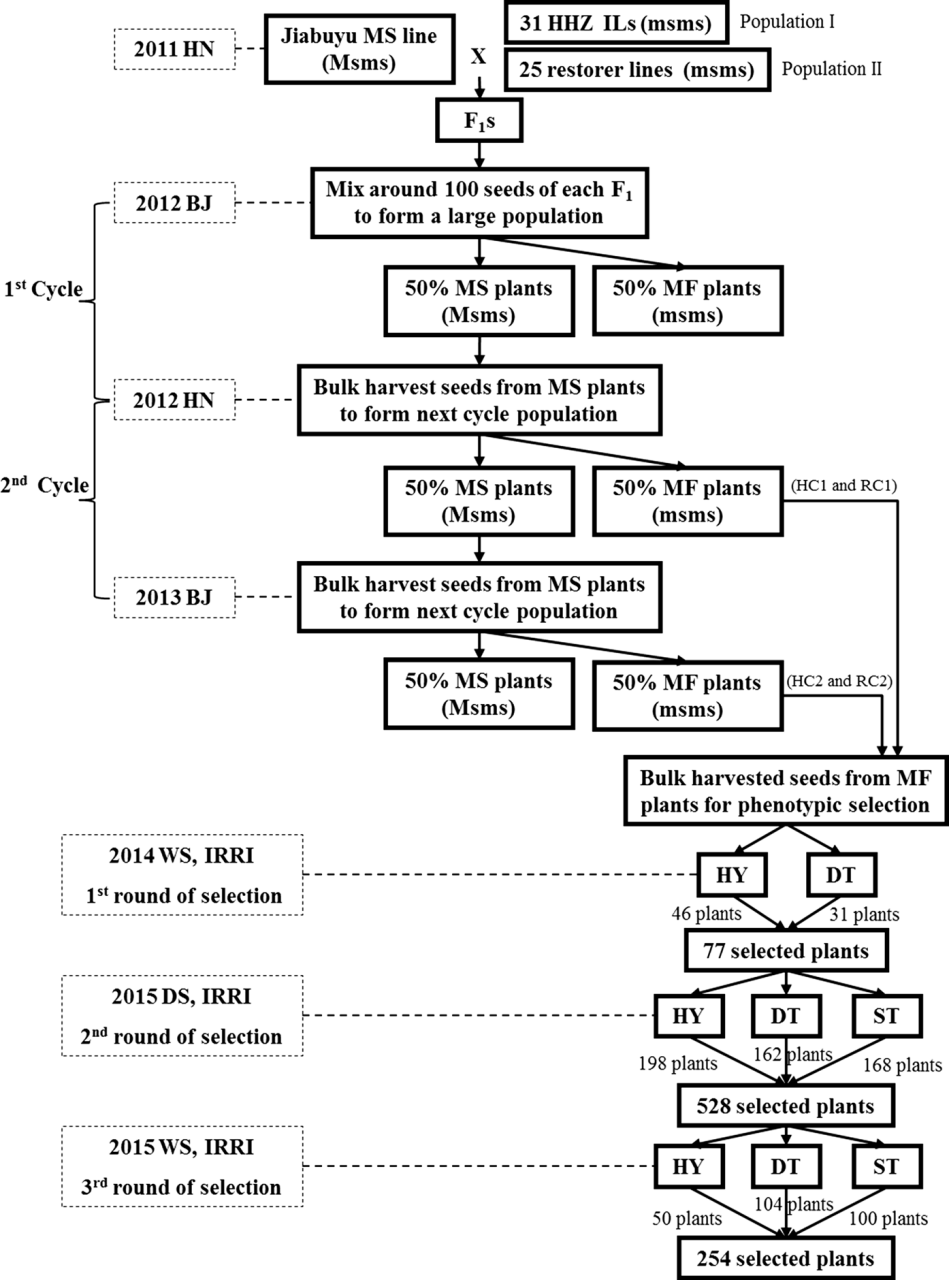
The breeding scheme for the development of recurrent selection populations facilitated by dominant male sterility. The founders of population I comprised 31 Huang-Hua-Zhan (HHZ) background introgression lines (ILs) and the founders of population II comprised 25 restorer lines applied in hybrid breeding. MS and MF indicated male sterile and male fertile. Breeding sites HN and BJ were Hainan and Beijing, China, respectively. HC1 and HC2 were bulk-harvested seeds from MF plants of population I in the 1st and 2nd cycle, and RC1 and RC2 were bulk-harvested seeds from MF plants of population II in the 1st and 2nd cycle. WS and DS were the wet season and dry season at IRRI, Los Baños, Philippines. HY, DT, and ST were selections carried out for high grain yield and drought- and salinity-tolerant plants under irrigated, drought stress, and salinity stress conditions, respectively

The materials consisted of four RS populations derived two each from two different sets of initial populations: the first was developed using 31 elite fixed introgression lines (ILs) in Huang-Hua-Zhan (HHZ) background as founder lines with significantly improved GY and drought and/or salt tolerance developed in our ongoing Green Super Rice breeding program (Table 1). These 31 promising founders were selected from 11 BC_2_F_2_ populations developed from crosses between HHZ and 11 diverse donors (CDR22, CR203, Gang46B, Khazar, IR64, IRAT352, OM1723, OM1706, PSBRC28, Teqing and Zhong413); the second was developed from 25 popular restorer lines applied in hybrid breeding (Table 1). DMS line ‘Jiabuyu’ controlled by a novel single dominant nuclear gene provided by the Sanming Agricultural Research Institute in Fujian of China, was used as the outcrossing facilitator to develop the initial populations. Figure 1 shows the strategy and the steps involved in the process of developing RS populations. These founders were crossed with ‘Jiabuyu’ in the 2011 winter season in Hainan (HN), China. For each of the two initial populations, around 100 F_1_ seeds from each cross were equally mixed and were planted in the 2012 summer season in Beijing (BJ). The field was isolated by a plastic sheet 2.5 m high to prevent possible pollen contamination from other rice materials. Before the onset of flowering, on a visual phenotypic basis, poor-looking plants were removed. During the flowering period, human-aided tripping was carried out to facilitate pollination under isolation. The DMS heterozygote (Msms) plants upon crossing with pollen fertile homozygote recessive (msms) plants resulted in a ratio of 1:1 MS and male fertile (MF) plants. The MS plants had the character of panicle enclosure. At maturity, the seeds of MS plants were bulk harvested and advanced to the next RS cycle. In the following two cycles, seeds from the MS plants were bulk harvested for the next round of recombination and seeds from MF plants were bulk harvested for screening. With this approach, we were able to develop four sets of RS populations with seeds from MF plants (HC1 and HC2 developed from RS cycle 1 and 2, respectively, from population I; RC1 and RC2 developed from RS cycle 1 and 2, respectively, from population II). The three standard check varieties included were NSIC Rc222, the high yield (HY) check for irrigated condition, and UPLRi7 and NSIC Rc184 as drought-tolerant (DT) and salt-tolerant (ST) checks, respectively.

**Table 1:** The founders of the two initial populations

Founders of population I		Founders of population II	
HHZ/CR203 BC2F5	HHZ11-DT7-SAL1-SAL-6	33	Mian-Hui725
HHZ/CR203 BC2F5	HHZ12 -DT10-SAL3	84	Ming-Hui63
HHZ/CR203 BC2F5	HHZ12-DT10-SAL1-DT1-1	611	Ming-Hui86
HHZ/CR203 BC2F5	HHZ12-Y7-DT2	9311	Min-Hui3301
HHZ/CR203 BC2F5	HHZ12-Y9-Y3	CDR22	Mi-Yang46
HHZ/CR203 BC2F5	HHZ17-SAL11-SAL4	Ce253	R9308
HHZ/CR203 BC2F5	HHZ19-DT-5-Y2	Cheng-Hui177	Shu-Hui527
HHZ/CR203 BC2F5	HHZ19-SAL14-SAL2	Duo-Xi 1 Hao	Wan-Hui057
HHZ/Gang46B BC2F5	HHZ2-Y3-Y1-SAL1	Fu838	Yan-Hui559
HHZ/IR64 BC2F5	HHZ3-DT6-LI1-LI1	Guang-Hui998	Yi-Hui1577
HHZ/OM1706 BC2F5	HHZ5-SAL8-DT2-SAL1-1	Gui99	Zhe-Hui7954
HHZ/OM1706 BC2F5	HHZ5-SAL8-DT3-SUB1-1	Hang 1 Hao	Zhong-Hui8006
HHZ/OM1706 BC2F5	HHZ5-Y8-Y2	Lu-Hui17	
HHZ10-SAL15-Y1-SAL3	HHZ6-DT7-LI1-LI1		
HHZ10-Y7-Y1-SAL2(-1)	HHZ6-Y2-Y1-DT1		
HHZ10-Y7-Y1-SAL2(-2)

### Selection schemes for improving GY and abiotic stress tolerance

The seeds of HC1, HC2, RC1, and RC2 were shipped to the International Rice Research Institute (IRRI) in the Philippines to perform phenotypic target trait screening, which included one round of single-plant selection for high GY under irrigated conditions and for DT in the initial generation, followed by two rounds of selection based on progeny testing of all selected lines across drought, salinity, and irrigated conditions as described below. At maturity, the promising HY, DT, and ST plants free of diseases were visually selected based on their overall superior agronomic performance (Fig. 1).

#### Normal irrigated conditions

Trials for HY were conducted in the irrigated field on the IRRI farm. Seeds were sown in the seedling bed in July in the wet season (WS) and in December in the dry season (DS), and 21-day-old seedlings were transplanted. The field was irrigated regularly and managed with standard crop management practices. The field received 160:60:60 kg of N:P:K fertilizer. Nitrogen was applied to irrigated plots at 160 kg/ha with five splits: 30 kg/ha basal; 30 kg/ha at 18 days after transplanting (DAT) to promote tillering; another 30 kg/ha at 33 and 43 DAT for panicle initiation, to promote spikelet differentiation; and finally, 40 kg/ha at 64 DAT for booting stage.

#### Drought stress conditions

Drought trials were conducted on the upland rice farms of the IRRI experiment station. Seeds were sown on the seedling bed in December in the DS and in July in the WS, and 21-day-old seedlings were transplanted into the flooded field. Then, irrigation was maintained at 5-cm depth for only 1 month. The field was then drained and irrigation was withheld completely until harvest. Thus, the plant materials were typically subjected to moderate (in the WS) to severe (in the DS) drought at the reproductive stage depending on the rainfall during the testing seasons. For the drought plots, 30:30:30 kg NPK fertilizer was applied for basal and 30 kg/ha of nitrogen was applied at 18 DAT and 33 DAT to fully match plant growth needs for nitrogen.

#### Salinity stress conditions

The salinity experiment was conducted in a farmer’s field with natural coastal salinity located in Infanta, Quezon, Philippines. Seeds were sown in the nursery seedbed in August for the WS and in January for the DS, and, later, 21-day-old seedlings were transplanted. Salinity levels were constantly monitored with an EM50 (Decagon Devices, Pullman, WA 99163) that ranged from 6.2 to 14.1 dS/m during the entire growth period from seeding to harvest depending upon the rainfall and high tides. Fertilizer of 120:40:40 kg N:P:K was applied with 50% of N and 100% of P and K as basal, 25% of N at active tillering (18 DAT), and 25% of N (33 DAT) near flowering to fully match plant growth needs.

### Replicated yield trials of the selected lines under stress and non-stress conditions

Work was done to evaluate GY and overall performance of the selected lines against the standard check varieties in order to identify superior lines to be nominated to national cooperative yield trials (NCYT) in different countries. These selected lines were evaluated along with three standard check varieties for their GY performance in replicated trials with similar planting space of 20 × 20 cm in a randomized complete block design separately under normal irrigated, drought, and salinity (Infanta, Quezon) stress conditions during the 2016 DS and 2016 WS at IRRI. The three checks were NSICRc222 (irrigated HY check), UPLRi7 (DT check), and NSICRc184 (ST check).

Under the three conditions described above (normal irrigated, drought stress, and salinity stress), seeds of selected lines and all three checks were sown in the seedling nursery in the 2016 DS and 25-day-old seedlings of each line and check were transplanted into a three-row plot with 12 plants per row. The plots were randomly arranged in the field with a spacing of 20 × 20 cm and two replications. All plots were recorded for their heading date when 50% of the plants started heading, and three representative plants within each plot were measured for their plant height and harvested at maturity for measuring GY (g/plant). Finally, we defined here lines having GY higher than that of the HY check (NSIC Rc222) in irrigated conditions, having GY higher than that of the DT check (UPLRi7) in drought conditions, and having GY higher than that of the ST check (NSIC Rc184) in salinity conditions as HY, DT, and ST lines, respectively. The promising HY, DT, and ST lines identified in the 2016 DS were planted again in the 2016 WS with larger plot size, 5 rows by 12 hills, and two replications in irrigated, drought, and salinity conditions. The middle 30 plants were bulk harvested to measure GY per plot (~ 1.2 m^2^) (g/plot).

### Data analysis

For the GY trials in the 2016 DS and WS in each of irrigated, drought, and salinity conditions, the best linear unbiased estimates (BLUE) of lines for GY were obtained using the PBTools package developed by IRRI (bbi.irri.org) by keeping the lines (genotype) and replicates as fixed effects. To estimate the variances of genotypes, replicates, and residuals, ANOVA was carried out using the “anova” function in R software (R Core Team 2015). Heritability (h^2^) was computed using the estimated variance components as MS_G_/(MS_G_ + MS_e_), where MS_G_ and MS_e_ are the mean square of genotypes and residuals, respectively.

## Results

### Selections for grain yield, drought and salinity tolerances

Table 2 shows the results over three rounds of selection. In the 2014 WS, 2000 single plants of each population were planted in irrigated and drought conditions. In total, 46 and 31 plant were selected under normal irrigated and drought stress condition with selection intensity (SI) being 0.58% and 0.39%, respectively. In 2015 DS, the above 77 plants from the first round of selection were progeny tested across all three test environments (irrigated, drought, and salinity) with a plot size of 5 rows by 12 hills for each selected plant, in which the second round of selection was practiced in order to first select good lines under each condition and then select 1–5 best-performing plants from each selected line. Under normal irrigated, drought and salinity conditions, 198 (SI = 4.29%), 162 (SI = 3.51%) and 168 (SI = 3.64%) best yield-performing plants were visually selected based on overall phenotypic performance, respectively. Together, the second round of selection resulted in a total of 528 plants. In 2015 WS, all 528 plants from the second round of selection were progeny tested across all three test environments, with a plot size of 3 rows by 12 hills for each plant. In total, the third round of selection resulted in 254 plants including 50 (SI = 0.26%), 104 (SI = 0.55%) and 100 (SI = 0.53%) plants from normal irrigated, drought and salinity conditions, respectively (Table 2).

**Table 2:** The number and yield performance of selected plants from three rounds of selection under irrigated, drought, and salinity conditions for four recurrent selection populations

Season	Conditions		HC1	HC2	RC1	RC2	Total
2014WS	Irrigated	No.	16	9	15	6	46
		Mean	22.7	24.1	29.5	29.6	26.1
		Range	13.2–39.4	12.9–50.0	11.6–53.9	15.6–41.5	11.6–53.9
	Drought	No.	12	8	7	4	31
		Mean	33.8	29.2	29.3	38.8	32.3
		Range	22.3–50.5	17.3–42.4	17.1–41.6	32.8–44.0	17.1–50.0
2015DS	Irrigated	No.	84 (28)	53 (16)	39 (20)	22 (10)	198 (74)
		Mean	59.6	63.6	61.1	57.8	60.8
		Range	38.3–92.0	42.7–92.8	37.3–85.2	43.2–78.6	37.3–92.8
	Drought	No.	78 (24)	22 (8)	51 (15)	11 (5)	162 (52)
		Mean	8.6	8.0	8.1	8.5	8.4
		Range	3.6–21.4	5.4–12.7	3.1–18.1	5.3–12.7	3.1–24.1
	Salinity	No.	61 (26)	37 (17)	50 (22)	20 (10)	168 (75)
		Mean	52.4	54.6	51.6	52.3	52.7
		Range	20.6–77.4	25.1–78.8	22.6–76.0	20.2–73.7	20.2–78.8
2015WS	Irrigated	No.	13 (11)	19 (13)	12 (10)	6 (4)	50 (38)
		Mean	32.3	32.5	30.2	35.7	32.3
		Range	15.4–74.2	21.7–59.1	15.0–42.9	23.2–51.6	15.0–74.2
	Drought	No.	38 (28)	30 (21)	21 (17)	15 (12)	104 (78)
		Mean	21.7	17.4	21.6	21.7	20.4
		Range	8.1–49.7	7.8–29.8	9.5–36.1	13.7–49.1	7.8–49.7
	Salinity	No.	57 (57)	17 (17)	20 (20)	6 (6)	100 (100)
		Mean	11.8	10.9	11.3	6.3	11.2
		Range	5.7–29.3	4.2–33.7	6.6–30.0	4.7–9.7	4.2–33.7

The numbers in parentheses are those lines selected from the last round of selection

*No.* the number of selected plants, *mean* the average GY (in g) of selected plants, *range* the grain yield variation (in g) of selected plants

### Yield trials in the 2016 DS

**Fig. 2:**
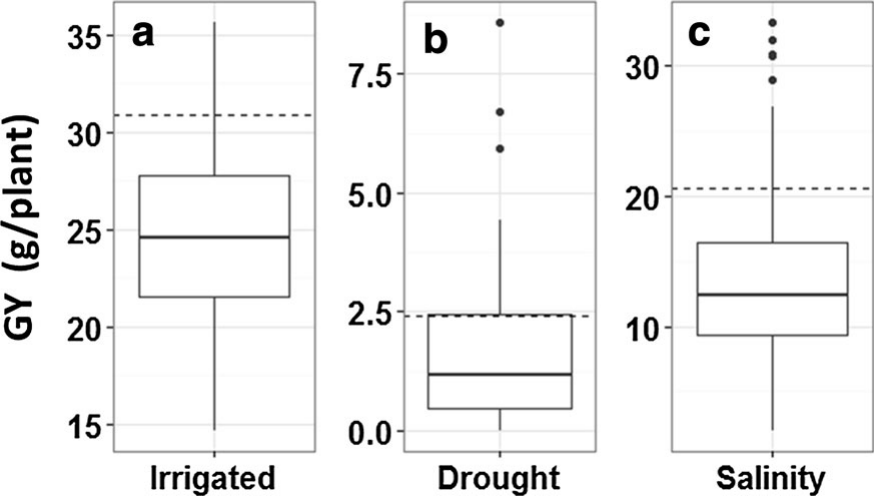
The GY (g/plant) distributions of 254 selected lines and checks in irrigated (**a**), drought (**b**), and salinity (**c**) conditions in the 2016 DS. The broken line indicates the GY of corresponding checks, including NSIC Rc222, UPLRi7, and NSIC Rc184, in irrigated, drought, and salinity conditions, respectively

ANOVA of the replicated trials carried out in three different (irrigated, drought, and salinity) conditions in the 2016 DS clearly showed the genotypes studied to be significantly different (P < 0.05). Yield trials conducted in the 2016 DS gave h^2^ of 0.60, 0.61, and 0.61 for GY under irrigated, drought, and salinity conditions, respectively (Table 3). The GY ranges of these 254 selected lines were 14.6–35.6, 0–8.6, and 2.0–33.3 g in irrigated, drought, and salinity conditions, respectively. The GY of Rc222 was 30.9 g in irrigated conditions. Among the 254 lines, 21 HY lines were found with GY ranging from 31.0 to 35.6 g. The GY of UPLRi7 was 2.43 g under drought stress, and 26 DT lines were found with GY ranging from 2.5 to 8.6 g. The GY of Rc184 was 20.6 g in salinity conditions. Among the 254 lines, 21 ST lines were identified with GY ranging from 21.0 to 33.3 g (Fig. 2).

**Table 3:** The ANOVA results for yield trials in irrigated, salinity, and drought conditions in the 2016 dry season (DS) and wet season (WS)

Season	Conditions	Source	Df	SS	MS	F	P	h^2^
2016 DS	Irrigated	Genotypes	253	10,398.3	41.1	1.5	0.0006	0.60
		Replicates	1	79.1	79.1	2.9	0.0897	
		Residuals	253	6893.6	27.2			
	Salinity	Genotypes	253	16,144.0	63.8	1.6	< 0.0001	0.61
		Replicates	1	59.1	59.1	1.5	0.2240	
		Residuals	253	10,057.4	39.8			
	Drought	Genotypes	253	402.6	1.6	1.6	0.0002	0.61
		Replicates	1	6.4	6.3	6.3	0.0128	
		Residuals	253	255.5	1.0			
2016 WS	Irrigated	Genotypes	42	428,688.0	10,206.9	2.4	0.0025	0.71
		Replicates	1	22,500.0	22,500.0	5.3	0.0257	
		Residuals	42	176,643.0	4205.8			
	Salinity	Genotypes	42	373,995.0	8904.6	1.8	0.0263	0.65
		Replicates	1	29,412.0	29,412.0	6.1	0.0181	
		Residuals	42	203,976.0	4856.6			
	Drought	Genotypes	42	207,037.8	4929.5	2.0	0.0144	0.67
		Replicates	1	22,268.7	22,268.7	9.0	0.0046	
		Residuals	42	104,350.5	2484.5

### Yield trials in the 2016 WS

**Fig. 3:**
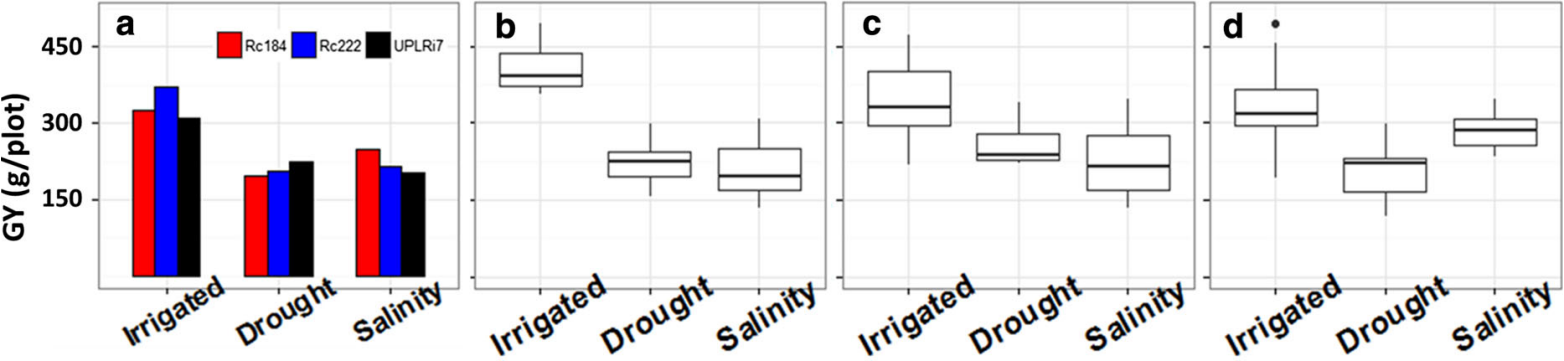
The GY (g/plot) of checks and selected lines in irrigated, drought, and salinity conditions in 2016 WS. **a** The GY of checks (NSIC Rc184, NSIC Rc222 and UPLRi7). **b** The GY of HY lines identified in the 2016 DS. **c** The GY of DT lines identified in the 2016 DS. **d** The GY of ST lines identified in the 2016 DS

The three checks and 43 lines, including 21 HY, 26 DT, and 21 ST lines, identified in the 2016 DS were planted again in the 2016 WS. Yield trials conducted in the 2016 WS gave h^2^ of 0.71, 0.65 and 0.67 for GY under irrigated, drought, and salinity conditions, respectively (Table 3). For the checks, in each condition, the corresponding check had higher GY than the other two checks. For instance, in irrigated conditions, the GY per plot of HY check Rc222 was 372.3 g, which was higher than that of UPLRi7 (309.1 g) and NSIC Rc184 (324.2 g) (Fig. 3 and Table 4).

**Table 4:** The identified DT, ST, and HY lines having GY significantly higher than that of the corresponding checks

Character	Pops	Designation	GY (g/plot)		
			Irrigated	Drought	Salinity
DT/ST/HY	HC1	IA-R11-D5-S1	453.3	297.9	308.0
DT/HY	HC1	IA-Y11-D5-S1	472.4	245.6	148.3
DT/HY	RC1	IC-Y14-D10-R1	403.1	297.2	216.1
DT/HY	RC1	IC-Y8-Y2-Y1	403.0	282.5	234.8
DT/ST	RC1	IC-Y4-Y1-R2	355.7	294.0	302.8
DT/ST	RC2	ID-Y5-S2-Y1	263.4	249.9	276.0
DT/ST	RC2	ID-Y5-Y1-Y2	335.2	291.1	346.4
HY	HC1	IA-Y3-Y3-S1	438.1	225.5	142.7
HY	HC1	IA-Y7-D8-S1	456.1	196.3	245.6
HY	HC2	IB-R7-Y3-R1	540.0	214.5	256.5
HY	HC2	IB-Y6-Y8-Y3	417.4	176.1	196.1
HY	RC1	IC-Y13-D4-R1	445.5	224.5	268.7
HY	RC1	IC-Y13-S1-S1	392.5	242.0	181.8
HY	RC2	ID-Y3-Y2-R1	422.8	228.2	132.6
DT	HC1	IA-R1-D2-R1	233.4	340.8	153.0
DT	HC1	IA-Y7-D9-Y1	328.5	248.2	149.3
DT	HC2	IB-R7-Y2-R1	301.2	263.7	168.3
DT	RC2	ID-Y5-S1-R3	218.3	272.4	184.9
DT	RC2	ID-Y5-Y1-R1	294.0	296.7	223.1
ST	HC1	IA-Y10-D7-R1	277.2	223.8	319.0
ST	HC1	IA-Y10-D7-Y1	190.7	197.9	286.5
ST	HC2	IB-Y1-S2-R1	366.4	118.2	292.2
ST	RC1	IC-Y4-Y1-Y1	295.2	222.6	314.2
ST	RC2	ID-Y5-S1-Y2	295.7	165.2	313.8
ST	RC2	ID-Y5-S2-R1	275.9	231.5	291.7
ST	RC2	ID-Y5-Y1-R2	316.4	205.6	310.2
ST	RC2	ID-Y5-Y1-Y3	287.8	152.0	287.7
HY check		Rc222	372.3	204.2	213.1
DT check		UPLRi7	309.1	225.1	203.7
ST check		Rc184	324.2	196.8	247.5

For the 21 HY lines, in irrigated conditions, 11 lines had GY ranging from 392.5 to 495.0 g, which was higher than that of NSIC Rc222 (372.3 g). The GY of the remaining 10 lines ranged from 355.7 to 378.3 g, which was similar with that of NSIC Rc222 (372.3 g). In drought and salinity conditions, the variation of GY was 155.4–297.9 and 132.6–308.0 g, respectively. Among 26 DT lines, 12 lines had GY ranging from 248.2 to 340.8 g in drought conditions, which was higher than that of UPLRi7 (225.1 g). The remaining 14 lines had GY ranging from 221.6 to 242.0 g, which was similar with that of UPLRi7 (225.1 g). In irrigated and salinity conditions, the ranges of GY were 218.3–474.0 and 132.6–346.7 g, respectively. For 21 ST lines, 12 lines had GY ranging from 274.7 to 346.7 g, which was higher than that of Rc184 (247.5 g). The remaining 10 lines had GY ranging from 234.8 to 274.7 g, similarly with that of Rc184 (247.5 g). In irrigated and drought conditions, the GY of these ST lines ranged from 190.7 to 495.0 g and from 118.2 to 297.9 g, respectively (Fig. 3 and Table 4).

## Discussion

### Dominant male sterility-facilitated recurrent selection

In the present study, DMS line ‘Jiabuyu’ was used to facilitate random outcrossing to develop the initial breeding population for RS breeding. In rice, the MS genes used for RS included cytoplasmic MS genes, photoperiod-/temperature-sensitive MS genes, recessive nuclear MS genes, and DMS genes (Quan et al. 2007; Er et al. 2004; Wang et al. 2001; Li and Zhang 2013). However, when using cytoplasmic MS genes, the parents should have the ability to restore fertility. The limited resources of cytoplasmic MS lines and restorer lines restrict the application of cytoplasmic male sterility in RS breeding. For photoperiod-/temperature-sensitive MS genes, their expression is affected by photoperiod and/or temperature, which restricts their application to environments. For recessive nuclear MS, on the one hand, the MS character cannot be presented in F_1_ and needs one time of selfing; on the other hand, the fertility of MF plants in recurrent recombinant populations includes homozygotes and heterozygotes, so that these MF plants cannot be directly used as breeding materials. One time of selfing is needed to identify homozygous MF plants, which would extend the breeding cycle (Virmani et al. 1997). For all three of these kinds of MS genes, the frequency of MS plants is low in RS populations, which would reduce the efficiency of outcross and recombination. DMS is the ideal one for RS breeding. ‘Jiabuyu’ is controlled by a single DMS gene (Yang et al. 2012). The genotypes of MS and MF plants are Msms and msms, respectively. In their hybrid offspring, the ratio of MS and MF plants is 1:1. This means that the MS plant could be present in the first generation with a high frequency (~ 50%), which could enhance the recombination efficiency. Further, the fertile MF plant is a homozygote, so that the MF plants could be directly used as breeding materials for trait screening. The RS population developed through a single DMS material has the advantageous characteristics of both cross-pollinated crops and self-pollinated crops. MS plants in the RS populations are cross-fertilized by MF plants, thus facilitating random recombination, whereas the MF plants are self-fertilized, thus increasing homozygosity. The simple RS breeding procedure could be easily handled by breeders. Further, new founder lines could be incorporated after a few years to widen the genetic base and incorporate important valuable traits. Therefore, DMS has considerable advantages, especially in RS breeding.

### Breeding rice cultivars with multiple stress tolerance

Here, the targeted breeding environments included abiotic stress conditions such as drought and salinity and normal irrigated conditions for screening for high GY. Two different kinds of RS populations were developed. For the first one, its founders comprised 31 ILs with improved salinity and drought tolerance and GY. Using this population, we aimed to breed further improved inbred lines by pyramiding multiple favorable QTLs through RS. For the other RS population, its founders comprised 25 restorer lines. We wanted to develop improved salt- and drought-tolerant restorer lines for the hybrid breeding program. Increasing rice yield potential under irrigated conditions or its yield under stress conditions is the most challenging breeding task (Ali et al. 2017). As many of the abiotic stress conditions are not a regular occurrence, modern varieties need to perform well under both favorable and unfavorable conditions. Directed selection and cross selection in targeted environments have proven to be a successful breeding strategy in the Green Super Rice breeding program to breed new materials that could tolerate more than one abiotic stress condition and produce high GY under normal favorable conditions as well (Ali et al. 2017).

The check NSIC Rc222, a modern rice variety released in the Philippines in 2010, has high GY in normal irrigated conditions. But, its yield in drought and salinity conditions is relatively lower than that of the corresponding checks (UPLRi7 and NSIC Rc184). Therefore, selecting lines with higher GY than UPLRi7 under drought and NSIC Rc184 under salinity is a reasonable approach to breeding cultivars that are better than Rc222 in stress conditions. During the process of single-plant selection, many plants had extremely high GY (Table 2) that may be caused by border effect. To obtain more reliable yield data, the selected plants were planted in a larger plot size in the 2016 WS. Finally, we identified 11 lines having significantly higher GY than Rc222 in irrigated conditions, which indicated that they had high yield potential. Additionally, 12 and 12 lines having significantly higher GY than the DT check (UPLRi7) and ST check (Rc184) in drought and salinity conditions were identified, respectively, indicating that these abiotic stress-tolerant lines have improved GY under stress conditions (Table 4).

Among these lines, IA-R11-D5-S1 had significantly higher GY than the corresponding checks across all conditions, indicating that this line had HY potential as well as improved GY in both drought and saline conditions. Its GY was 453.3, 297.9, and 308.0 g in irrigated, drought, and salinity conditions, respectively (Table 4). Three lines, IA-Y11-D5-S1, IC-Y14-D10-R1, and IC-Y8-Y2-Y1, had significantly higher GY than the corresponding checks in both irrigated and drought conditions, indicating that these three lines had HY potential as well as improved yield in drought condition. The GY was 472.4 g (245.6 g), 403.1 g (297.2 g), and 403.0 g (282.5 g) in irrigated (drought) condition for IA-Y11-D5-S1, IC-Y14-D10-R1 and IC-Y8-Y2-Y1, respectively (Table 4). Three lines, IC-Y4-Y1-R2, ID-Y5-S2-Y1 and ID-Y5-Y1-Y2, had higher GY in drought and salinity conditions than their corresponding checks, suggesting that these three lines had improved GY in both stress conditions. The GY was 294.0 g (249.9 g and 291.1 g) in drought and 302.8 g (276.0 g and 346.4 g) in saline conditions for IC-Y4-Y1-R2 (ID-Y5-S2-Y1 and ID-Y5-Y1-Y2). Among the three lines, IC-Y4-Y1-R2 yielded 355.7 g in irrigated conditions, which is similar to that of NSIC Rc222 (372.3 g), indicating that this line didn’t lose much yield under normal irrigated conditions (Table 4).

All the remaining seven HY lines (except IB-Y6-Y8-Y3) had GY similar to that of the corresponding checks in at least one of either drought or salinity conditions, which suggested that these lines had HY potential in normal irrigated conditions, but didn’t lose yield in at least one of either drought or salinity stress conditions (Table 3). One of the remaining ST lines (IB-Y1-S2-R1) had yield similar to that of NSIC Rc222 under irrigated conditions, indicating that this line had high yield under saline conditions but didn’t lose GY under normal conditions (Table 4).

### Critical considerations for adopting a DMS-facilitated RS approach

The initial population comprised dozens of founders, so the size of the population used for screening should be large enough to make sure promising recombinants could appear. Usually, for conventional breeding populations involving two parents, the size of the population used for screening is around 300. But, here, for RS, the population size for initial screening is 2000 to make sure we can find the best recombinants. In theory, as the population is large enough, the best expected recombinant could be present in the first recombination generation. During the screening process, we also observed that the performance between HC1 and HC2 or RC1 and RC2 was similar, which indicated that the second recombination generation didn’t generate an advantage over the first one. The possible reasons are that (1) the recombination efficiency of the DMS RS population is quite high, so that one time of random mating could break linkage drag; (2) the population applied for trait screening is large enough, so that promising recombinants could be present.

In total, 27 DT, ST and/or HY lines were identified, consisting of 8, 4, 6 and 9 from HC1, HC2, RC1 and RC2 populations, respectively (Table 4). As the lines from RC1 and RC2 were derived from restorer lines, these lines could be applied in hybrid breeding to develop high GY hybrids with improved drought and salinity tolerance. The combining ability testing of these newly developed lines is ongoing in our breeding program.

Although the promising HY, DT and ST lines were identified from four RS population, the results were not as good as we had expected. We observed that the highest GY of HY lines did not have significant yield advantages when compared to NSIC Rc222. Also, the number of identified promising HY, DT and ST lines were not as high as one would expect. One of the main reasons might be the genetic background of the MS plants itself. In our RS breeding program, each founder was crossed with ‘Jiabuyu’, and then the F_1_ seeds were equally mixed. In the offspring, the genotypic frequency of ‘Jiabuyu’ is always around half, so the performance of MS material is quite important and needs to be in the most adaptable background, especially for the tropics. However, in our case, Jiafuzhan is a high-quality early *Xian* variety developed by Xiamen University and released in 2003 in Fujian, China, especially for sub-temperate conditions. Jiafuzhan heads in 123 days, with a plant height of 105 cm, nine panicles per plant, with a high fertility rate of 90%, and the flag leaf is short and straight. The agro-morphological features of Jiafuzhan showed relatively poor adaptation to the tropical environments of South and Southeast Asia, which might have resulted in the overall reduced performance of the RS populations. To resolve this problem, we are transferring the DMS gene into other elite varieties that are highly adaptable to the tropics such as HHZ (officially released in Mozambique, Indonesia, and India) and Hua-Zhan. Another solution to resolve the genetic background effect is to transfer the DMS genes into all the founders. We can obtain DMS materials with various backgrounds. These seeds could be equally mixed, so that, in this type of RS population, each founder line has equal genotype frequency.

Another problem that we observed was low fertility rate in some of the MF plants, which showed in one or two panicles to possess nearly half of the spikelets to remain completely sterile, which we understand might have been influenced by the DMS gene. However, this problem was not so frequent and was resolved by restricting selection to eliminate such low-fertility plants.

## Conclusion

MS line governed by single DMS gene was an ideal tool for rice RS breeding. Through this approach, we identified 11 promising HY lines under irrigated condition, 12 DT and 12 ST lines. Among them, one line (IA-R11-D5-S1) gave higher grain yield across all three conditions, three lines (IA-Y11-D5-S1, IC-Y14-D10-R1 and IC-Y8-Y2-Y1) yielded high in both irrigated and drought conditions and another three lines (IC-Y4-Y1-R2, ID-Y5-S2-Y1 and ID-Y5-Y1-Y2) gave high yields in both drought and salt-stressed conditions. Our breeding practices conducted in the present study provided valuable lessons for other rice breeders. The genetic background of MS line, the breeding approach to develop initial RS population and population size for trait screening are important considerations for the success of RS breeding. The developed elite lines are promising to be high yield, drought and/or salinity tolerant rice varieties.

## Abbreviations

BJ: Beijing
BLUE: Best linear unbiased estimates
DAT: Days after transplanting
DMS: Dominant male sterile
DS: Dry season
DT: Drought-tolerant
GY: Grain yield
HHZ: Huang-Hua-Zhan
HN: Hainan
HY: High yield
ILs: Introgression lines
MF: Male fertile
MS: Male sterile
RS: Recurrent selection
ST: Salt-tolerant
WS: Wet season
